# Grape Polyphenols Ameliorate Muscle Decline Reducing Oxidative Stress and Oxidative Damage in Aged Rats

**DOI:** 10.3390/nu12051280

**Published:** 2020-04-30

**Authors:** Giuseppe Annunziata, Manuel Jimenez-García, Silvia Tejada, David Moranta, Angela Arnone, Roberto Ciampaglia, Gian Carlo Tenore, Antoni Sureda, Ettore Novellino, Xavier Capó

**Affiliations:** 1 Department of Pharmacy, University of Naples Federico II, Via Domenico Montesano 49, 80131 Naples, Italy; giuseppe.annunziata@unina.it (G.A.); roberto.ciampaglia@unina.it (R.C.); ettore.novellino@unina.it (E.N.); 2 Laboratory of Neurophysiology, Biology Department, University of Balearic Islands (UIB), Ctra. Valldemossa Km 7.5, E-07122 Palma de Mallorca, Spain; manuel.jimenez@uib.es (M.J.-G.); david.moranta@uib.es (D.M.); 3 Laboratory of Neurophysiology, Biology Department and Health Research Institute of the Balearic Islands (IdISBa), University of Balearic Islands, E-07122 Palma de Mallorca, Spain; silvia.tejada@uib.es; 4 CIBEROBN (Physiopathology of Obesity and Nutrition), Instituto de Salud Carlos III, 28029 Madrid, Spain; antoni.sureda@uib.es; 5 Dipartimento di Medicina Clinica e Chirurgia, Unit of Endocrinology, Federico II University Medical School of Naples, Via Sergio Pansini 5, 80131 Naples, Italy; angela.arnone15@gmail.com; 6 Research Group in Community Nutrition and Oxidative Stress and Health Research Institute of the Balearic Islands (IdISBa), University of Balearic Islands, E-07122 Palma de Mallorca, Spain; xavier.capo@uib.es

**Keywords:** ageing, skeletal muscle, polyphenols, nutraceutical, grape

## Abstract

A large number of studies have demonstrated the implication of oxidative stress (OxS) in the pathogenesis of ageing-related muscle decline and atrophy. The key mechanism is related to the OxS-induced production of free radicals, with the consequent increase in oxidative damage, resulting in affected muscle quality and strength. The present study aimed to evaluate the efficacy of a grape polyphenol-based nutraceutical formulation (Taurisolo^®^) in reducing the OxS in muscle of aged rats. A group of 16 aged (20 months) rats were orally administered with Taurisolo^®^ (*n* = 8; 100 mg/kg Taurisolo^®^) or placebo (*n* = 8; 50 mg/kg maltodextrin); an additional group of eight young (three months) rats were also treated with placebo. All the treatments were orally administered for 30 days. The activities of antioxidant enzymes, the levels of malondialdehyde (MDA) and nitrotyrosine (N-Tyr) and the expression of OxS- and inflammation-related genes were evaluated on the gastrocnemius muscle. In muscle samples of the treated-group, increased activity of antioxidant enzymes, reduced MDA and N-Tyr levels and increased expression of antioxidant and anti-inflammatory genes were observed in respect to the placebo. Data herein presented suggest that the chronic treatment with Taurisolo^®^ significantly reduces oxidative damage and improves muscle performance in aged rats.

## 1. Introduction

Ageing is a biological process characterized by a progressive functional decline involving the whole body [1]. A large number of studies have been conducted to highlight the mechanisms at the basis of this process and, among them, the increased oxidative status seems to play a central role. In particular, in 1968 Harman postulated the so-called Free radical theory indicating oxidative stress (OxS) as the major causal factor of senescence [2,3]. In particular, with advancing age, the endogenous antioxidant defences suffer a progressive decline, resulting in altered pro-/antioxidant balance and increased OxS. In turn, this could be a trigger for cell damage processes that cannot be counteracted by internal mechanisms, causing a drastic organ mass and functionality loss and culminating in system dysfunction [4,5].

Besides the internal organs, these orchestrated processes also involve muscles, which appears interesting since skeletal muscle has a central role in the systemic regulation of age-related diseases [6]. Indeed, growing evidence reported the implication of OxS in the pathogenesis of ageing-related muscle atrophy [7,8,9,10,11,12]. The key mechanism has been related to the OxS-induced production of free radicals, with the consequent increased oxidative damage such as lipid peroxidation, resulting in affected muscle quality and strength. In addition, the severely attenuated ability of aged skeletal muscle to counterbalance the increased production of reactive oxygen species (ROS) results in an oxidative damage accumulation, causing a loss of tissue homeostasis [13].

To support the relationship between OxS and age-related impairment of muscle quality, it was previously demonstrated that the production of ROS is higher in aged rats than in young ones [14]. In addition, circulating levels of OxS biomarkers in human were found to be elevated in elderly subjects with a diagnosis of sarcopenia [15,16], suggesting that OxS may contribute to age-related muscular decline.

Although the best strategy for the treatment of age-associated functional declines is regular exercise [17,18], seeking novel and eventually natural approaches may help reduce the risk of sarcopenia and functional loss. Hence, the need to find novel and eventually natural approaches is increasing. In this context, polyphenols are good candidates. Besides the historically known antioxidant activity exerted at different levels, intriguing evidence demonstrated the ability of polyphenols to prevent muscle impairment related to ageing, in particular contrasting the OxS [14]. In fact, various evidence showed the ability of resveratrol (RSV), one of the main polyphenols in grape, to prevent muscle atrophy in a large number of catabolic conditions [19,20,21,22], contrasting the muscular oxidative damage induced by ageing [23]. Moreover, a recent study demonstrated that, in aged rats, RSV is able to ameliorate muscle atrophy, contributing to the maintenance of mitochondrial function and reducing the OxS through the PKA/LKB1/AMPK pathway [24]. Furthermore, RSV has been reported to activate Sirtuin 1 (Sirt1) [25,26]—which has a beneficial role in various ageing-related conditions, including muscle mass and functional decline—and this link is well established [25]. Overall, this evidence elucidates the key mechanisms underlying the protective effects of this polyphenol in the prevention of age-related muscle decline.

Based on the available literature, the present study aimed to evaluate the efficacy of a novel grape polyphenol-based nutraceutical formulation (registered as Taurisolo^®^) in ameliorating the status of OxS markers as parameters of muscle quality in aged rats.

## 2. Materials and Methods

### 2.1. Taurisolo^®^ Supplement Preparation

Taurisolo^®^ supplement is a polyphenolic extract obtained from *Aglianico* cultivar grape, collected during the harvest in autumn 2016. The Department of Pharmacy, University of Naples Federico II (Naples, Italy), firstly formulated the supplement, and the MBMed Company (Turin, Italy) accomplished the large-scale production. Grapes were extracted with hot water (50 °C). The extract was then centrifugated and underwent a spray-drying process to obtain a fine powder microencapsulated formulation with maltodextrins (pomace:maltodextrins ratio 1:1, *w*/*w*). The polyphenol profile of Taurisolo^®^ was obtained by High-Performance Liquid Chromatography-diodearray detector (HPLC-DAD, Jasco Inc., Easton, MD, USA) analysis. The HPLC-DAD analysis indicated that Taurisolo^®^ is a polyphenol-rich extract. Among the main polyphenols contained in Taurisolo^®^: Ferulic acid 10.5 ± 0.70 µg/g, Resveratrol 13.6 ± 0.64 µg/g, Caffeic acid 20.7 ± 0.76 µg/g, Procyanidin B3 dimer 22.05 ± 6.61 µg/g, p-coumaric acid 27.9 ± 0.66 µg/g, Rutin 28.4 ± 0.70 µg/g, Quercetin 40.22 ± 7.11 µg/g, Procyanidin C2 trimer 44.6 ± 0.66 µg/g, Procyanidin B4 dimer 56.6 ± 0.88 µg/g, Procyanidin B1 dimer 62.8 ± 0.59 µg/g, Procyanidin B2 dimer 426.5 ± 5.92 µg/g, Syringic acid 539.2 ± 6.02 µg/g, Epicatechin 886.0 ± 7.82 µg/g, Gallic acid 1463.4 ± 65.5 µg/g; Catechin 4087.0 ± 64.5 µg/g [27].

### 2.2. Animals//Ethics Approval

Old male Sprague-Dawley rats (20 months; 580 ± 11.8 5 g weight; *n* = 32; Charles River Laboratories, Barcelona, Spain) were housed individually in standard cages under controlled environmental conditions (20 ± 2 °C; 70% humidity, and 12-h light/dark cycle, lights on at 08:00) with free access to standard food (Panlab A04, Panlab S.L.U., Barcelona, Spain) and tap water. All procedures were performed during the light period and in accordance with the European Convention for the Protection of Vertebrate Animals used for Experimental and other Scientific Purposes (Directive 86/609/EEC) and approved by the Bioethical Committee of the University (approval file number 2019/14/AEPX).

### 2.3. Experimental Design

The animals were chronically treated once daily for 30 days. The aged placebo group (*n* = 8) and the young control group (*n* = 8) orally received 50 mg/kg of maltodextrin (Sigma–Aldrich, Madrid, Spain) as a vehicle, and the aged rats (*n* = 8) were orally treated with 100 mg/kg of Taurisolo^®^. For the treatments, both Taurisolo^®^ or maltodextrin were separately dissolved in water obtaining 100 mg/mL solutions that were orally administered, based on the animal body weights, in order to reach the treatment doses. Before starting the treatments, all the animals were accustomed to both the solution flavour and the mode of administration with 1–2 mL of maltodextrin solution for a week. This preventive procedure allowed high animal compliance for the 30-day treatment. All rats were sacrificed by decapitation 30 days after the treatment beginning at 08:00 (during dark/light change). Gastrocnemius muscles were quickly removed, immediately frozen in liquid nitrogen, and stored at −80 °C until analysis.

### 2.4. Motor Performance and Coordination in Rotarod Test

Motor performance and balance were evaluated by means of a rotarod (Panlab^®^). Animals performed training sessions during five days prior to the test (one session/day) on the rotarod at a constant speed (4 rpm) until they attained a stable performance. On the test day, the rats were placed on the rotarod in acceleration mode (from 4 to 40 rpm over a period of 60 s) in order to evaluate their latency to fall down. Each rat repeated the test five times, leaving some minutes for recovery between tests. The mean measured was used as the motor coordination value. The rotarod design was performed at the beginning of the treatments (t0) and after the 30 days of the treatments (t30).

### 2.5. Gastrocnemius Muscle Homogenate

Gastrocnemius muscle portions (100 mg) were homogenized in a relationship 1:5 in a solubilization buffer (250 mM sucrose, 20 mM Tris–HCl, 40 mM KCl, and 2 mM EGTA, pH 7.4), using a disperser (IKA T10 basic ULTRA-TURAX). The homogenates were sonicated at 20 W and centrifuged (at 5000 *g*, 4 °C, for 15 min) and supernatants were stored at −80 °C until their utilization. Total protein content was measured by Bradford’s protein–dye binding assay [28].

### 2.6. Total Antioxidant Capacity (FRAP)

The total antioxidant capacity was measured using the ferric reducing antioxidant power (FRAP) assay, following the method described by Benzie and Strain [29].

### 2.7. Antioxidant Activities Determination

Catalase (CAT), superoxide dismutase (SOD), glutathione reductase (GRd) and glutathione peroxidase (GPx) activities were determined in gastrocnemius muscle homogenates. All activities were determined with a Shimadzu UV-2100 spectrophotometer at 37 °C. CAT activity was measured by the spectrophotometric method of Aebi based on the decomposition of H_2_O_2_ [30]. SOD activity was measured by an adaptation of the method of McCord and Fridovich [31]. GRd activity was measured by a modification of the Goldberg and Spooner spectrophotometric method [32]. GPx activity was measured using an adaptation of the spectrophotometric method of Flohe and Gunzler [33]. Results were normalized with protein concentration.

### 2.8. Malondialdehyde Assay

Malondialdehyde (MDA) as a marker of lipid peroxidation was analyzed in gastrocnemius muscle homogenate by a colorimetric assay based on the reaction of MDA with a chromogenic reagent to yield a stable chromophore with maximal absorbance at 586 nm. Briefly, samples or standards were placed in glass tubes containing n-methyl-2-phenylindole (10.3 mM) in acetonitrile:methanol (3:1). HCl 12 N was added, and the samples were incubated for 1 h at 45 °C. The absorbance was measured at 586 nm.

### 2.9. N-Tyrosine Determination

Nitrotyrosine (N-Tyr) was determined by the immunological method OxiSelect™ Nitrotyrosine Immunoblot Kit (Cell Biolabs, INC) following the manufacturer’s instructions. Total protein concentrations were measured by the method of Bradford [28]. Ten micrograms of protein from homogenate samples were transferred onto a nitrocellulose membrane by the dot blot method. Nitrocellulose membranes were incubated with rabbit anti-N-Tyr antibody. This step was followed by incubation with a horseradish peroxidase antibody (goat anti-rabbit IgG) conjugate directed against the primary antibody. The membrane was then treated with luminol, which is converted to a light-emitting form at wavelength 428 nm by the antigen/primary antibody/secondary antibody/peroxidase complex. The light was visualized and detected by short exposure to a Chemidoc XRS densitometer (Bio-Rad Laboratories, Alcobendas, Madrid, Spain). Image analysis was performed using Quantity One-1D analysis software (Bio-Rad Laboratories, Alcobendas, Madrid, Spain). The coefficient of variation has been calculated to be 12% for N-Tyr index.

### 2.10. Gene Expression

Total RNA was obtained from 0.1 g from gastrocnemius muscle using TriPure Isolation Reagent (Roche Diagnostics, Mannheim, Germany) following the manufacturer’s instructions. Total RNA was quantified using the Take3 Microplate in a PowerwaveXS spectrophotometer (BioTek, Winooski, VT, USA). A 1-μg sample of total RNA was reverse transcribed to cDNA using 25 U MuLV reverse transcriptase in a 5-μL retrotranscription mixture (10 mM Tris–HCl pH 9.0, 50 mM KCl, 0.1, 2.5 mM MgCl2, 2.5 μM random hexamers, 10 U RNase inhibitor, and 500 μM of each dNTP) for 60 min at 42 °C in a Gene Amp 9700 thermal cycler (Applied Biosystems, Alcobendas, Madrid, Spain). cDNA solutions were diluted 1/10, and aliquots were frozen (−20 °C) until analyzed. Real-time PCR was carried out using SYBR Green technology in a LightCycler rapid thermal cycler (Roche Diagnostics, Mannheim, Germany). The amplification program consisted of a preincubation step for denaturation of template cDNA (95 °C, 10 min) followed by 45 cycles consisting of a denaturation, an annealing, and an extension step under the conditions given in Table 1. After each cycle, fluorescence was measured at 72 °C.

### 2.11. Statistical Analyses

Statistical analysis was carried out using the Statistical Package for Social Sciences (SPSS v.21.0 for Windows). Results are expressed as mean ± SEM, and *p* < 0.05 was considered statistically significant. A Shapiro–Wilk test was applied to assess the normal distribution of the data. When the data were normally distributed, statistical significance was assessed by one-way analysis of variance (ANOVA) depending on the sample analyzed. The Spearman correlation coefficients was used to analyze associations between Rotarod permanence time at t30 and OxS- and oxidative damage-related markers. Levels of significance was set at *p* ≤ 0.05.

## 3. Results

### 3.1. Animal Body Weight

**Figure 1:**
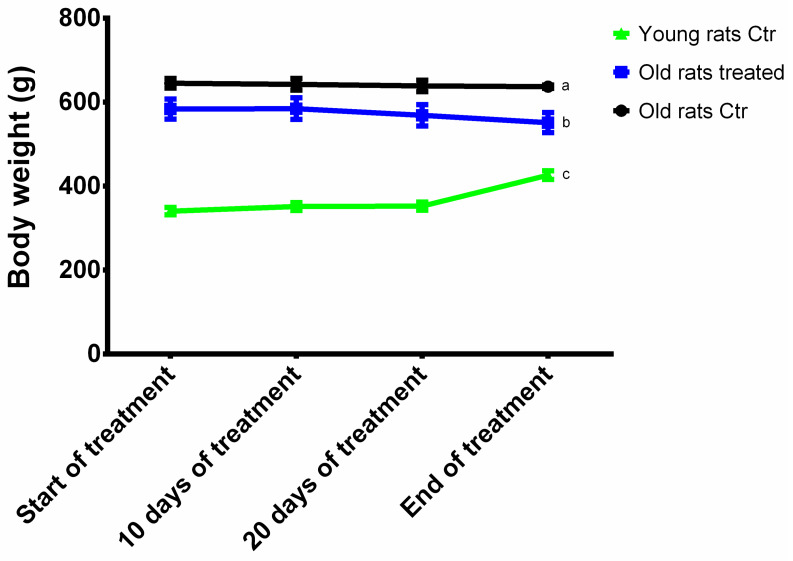
Body weight variations. Results are expressed as mean ± SEM, and *p* < 0.05 was considered statistically significant. Statistical significance was calculated by one-way ANOVA followed by Bonferroni’s post-hoc test. Different letters reveal significant differences. Old rats Ctr refers to old animals treated with placebo; Old rats treated refers to old animals treated with Taurisolo^®^; Young rats Ctr refers to young animals treated with placebo. T0 refers to the beginning of the treatment and t30 refers to the end of the treatment.

Animals were weighted at t0, t30 and during the treatment. The body weight variations of each animal group are reported in Figure 1. At t0, young rats weighted less (340.55 ± 8.87 g) than old rats (647.27 ± 11.60 g and 583.70 ± 24.15 g, Ctr and treated, respectively), and the body weight increased at t30 (426.35 ± 10.46 g). On the contrary, at the end of the treatment the old rats body weight reduced (637.00 ± 4.14 g and 551.45 ± 23.80 g, Ctr and treated, respectively).

### 3.2. Taurisolo^®^ Treatment Ameliorates the Motor Performance and Coordination

**Figure 2:**
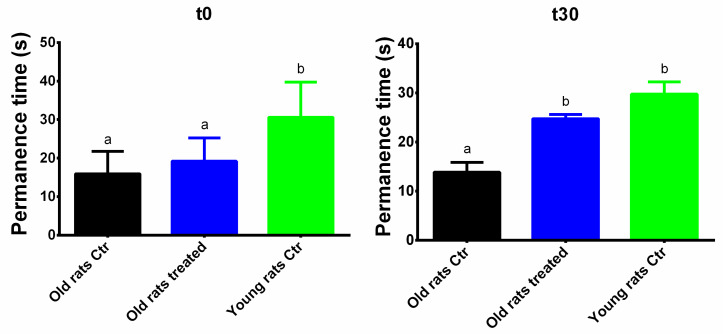
Motor performance and coordination. Permanence time evaluated with the Rotarod test before (t0) and after treatment (t30). Results are expressed as mean ± SEM, and *p* < 0.05 was considered statistically significant. Statistical significance was calculated by one-way ANOVA followed by Bonferroni’s post-hoc test. Different letters reveal significant differences. Old rats Ctr refers to old animals treated with placebo; Old rats treated refers to old animals treated with Taurisolo^®^; Young rats Ctr refers to young animals treated with placebo. T0 refers to the beginning of the treatment and t30 refers to the end of the treatment.

Motor performance and coordination was evaluated with the Rotarod test, taking into account the permanence time on the Rotarod during the five repeated sessions (Figure 2). At t0, the permanence time between the two groups of aged rats was not significantly different (15.94 ± 6.27 s and 19.20 ± 7.23 s, for control old rats and treated old rats, respectively). On the contrary, the permanence time of young rats was significantly higher (30.85 ± 10.74 s, *p* < 0.01). At t30, the permanence time (i) did not significantly decrease in the aged control group (13.86 ± 5.33 s) and (ii) significantly increased in the treated old rats (24.75 ± 2.44 s, *p* < 0.05); in this last group, the observed results are still significant when compared to the aged control (*p* < 0.01). Interestingly, although the permanence time at t0 was significantly different between the old treated and young groups (*p* < 0.01), at the end of the treatment no significant differences were observed.

### 3.3. Taurisolo^®^ Treatment Increases the Total Antioxidant Capacity and the Activity of Antioxidant Enzymes in Muscle

**Figure 3:**
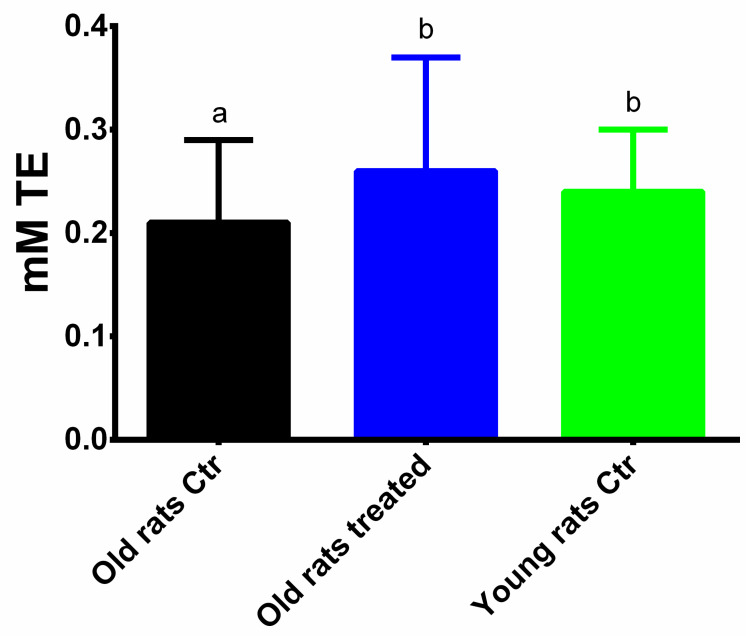
Antioxidant capacity determined with the FRAP assay in muscles of rats. Results are expressed as mean ± SEM, and *p* < 0.05 was considered statistically significant. Statistical significance was calculated by one-way ANOVA analysis. Different letters reveal significant differences. Old rats Ctr refers to old animals treated with placebo; Old rats treated refers to old animals treated with Taurisolo^®^; Young rats Ctr refers to young animals treated with placebo.

The antioxidant capacity was evaluated using the FRAP assay. As shown in Figure 3, in old rats treated with placebo the antioxidant capacity was significantly lower than in young rats (0.21 ± 0.08 mM Trolox Equivalents (TE) and 0.24 ± 0.06 mM TE, respectively; *p* < 0.05). On the other hand, in old rats treated with Taurisolo^®^ the antioxidant capacity was significantly higher than in the old rats control (0.26 ± 0.11 mM TE, *p* < 0.05). Interestingly, no significant differences were observed between old rats treated with Taurisolo^®^ and young rats.

**Figure 4:**
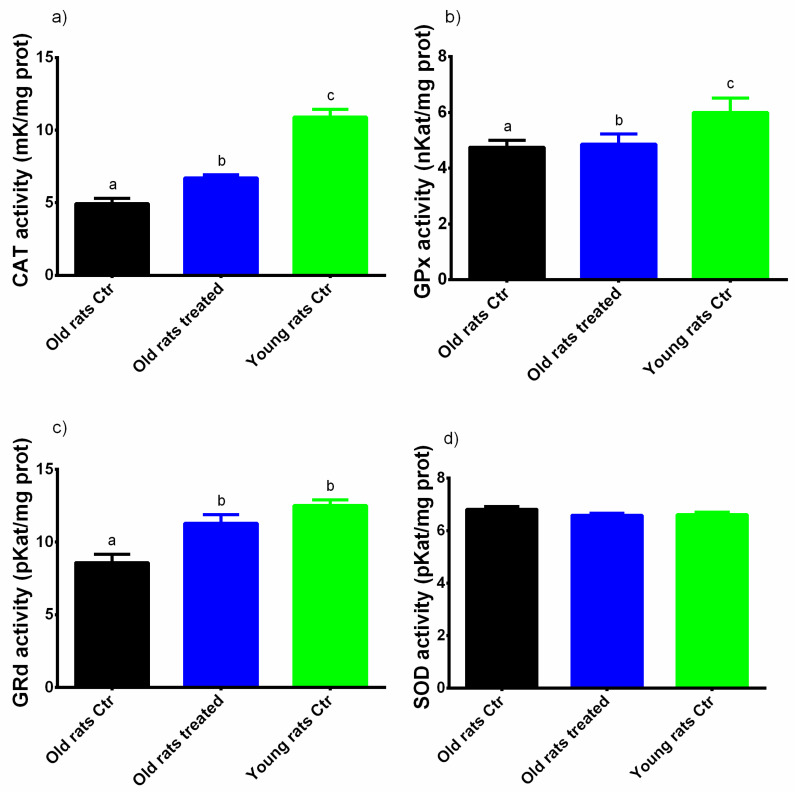
Enzymatic activity of (**a**) CAT, (**b**) GPx, (**c**) GRd and (**d**) SOD. Results are expressed as mean ± SEM, and *p* < 0.05 was considered statistically significant. Statistical significance was calculated by one-way ANOVA analysis. Different letters reveal significant differences. Old rats Ctr refers to old animals treated with placebo; Old rats treated refers to old animals treated with Taurisolo^®^; Young rats Ctr refers to young animals treated with placebo. **Abbreviations:** GRd, glutathione reductase; GPx, glutathione peroxidase; CAT, catalase; SOD, superoxide dismutase.

The enzymatic activities of CAT, SOD, GPx and GRd are presented in Figure 4. In the old rats control, the activities of antioxidant enzymes were lower than in young rats (GRd = Old rats control: 8.59 ± 0.57 pKat/mg prot, Young rats control: 12.50 ± 0.41 pKat/mg prot; GPx = Old rats control: 4.75 ± 0.25 nKat/mg prot, Young rats control: 5.99 ± 0.52 nKat/mg prot; CAT = Old rats control: 4.94 ± 0.36 mKat/mg prot, Young rats control: 10.90 ± 0.54 mKat/mg prot). No differences in SOD activity were observed between the different groups. Interestingly, in animals treated with Taurisolo^®^ the activities of GRd, GPx and CAT were significantly higher than in old rats treated with placebo (GRd = 11.30 ± 0.59 pKat/mg prot; GPx = 4.86 ± 0.37 nKat/mg prot; CAT = 6.70 ± 0.22 mKat/mg prot, *p* < 0.05 for all).

In order to underline the relationship between OxS and muscle performance, we analyzed the correlations existing between OxS-related markers and motor performance parameters evaluated with Rotarod test. In particular, significant positive correlations have been found between Rotarod test permanence time at t30 and CAT (0.68, *p* = 0.01) and GRd (0.49, *p* < 0.05) activities.

### 3.4. Taurisolo^®^ Treatment Reduces the Levels of MDA and N-Tyrin Muscle

**Figure 5:**
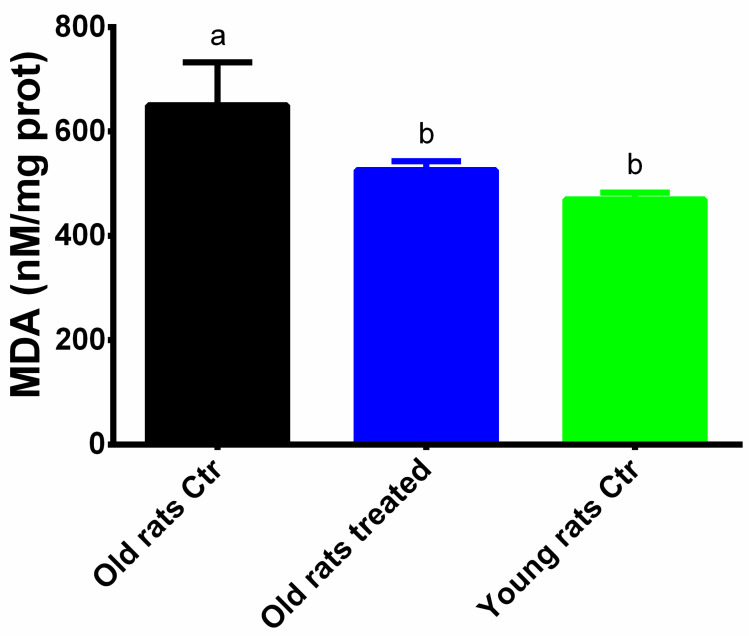
Levels of MDA. Results are expressed as mean ± SEM, and *p* < 0.05 was considered statistically significant. Statistical significance was calculated by one-way ANOVA analysis. Different letters reveal significant differences. Old rats Ctr refers to old animals treated with placebo; Old rats treated refers to old animals treated with Taurisolo^®^; Young rats Ctr refers to young animals treated with placebo. **Abbreviations:** MDA, malondialdehyde.

Lipid peroxidation as a marker of oxidative damage to lipids was evaluated measuring the levels of MDA. As shown in Figure 5, chronic treatment with Taurisolo^®^ counteracts significantly the increase in MDA levels induced by ageing (650 ± 82.7 mM/mg prot vs. 526 ± 16.7 mM/mg prot, Old rats control vs. Old rats treated, *p* < 0.05; Young rats control: 470 ± 13.1 mM/mg prot).

**Figure 6:**
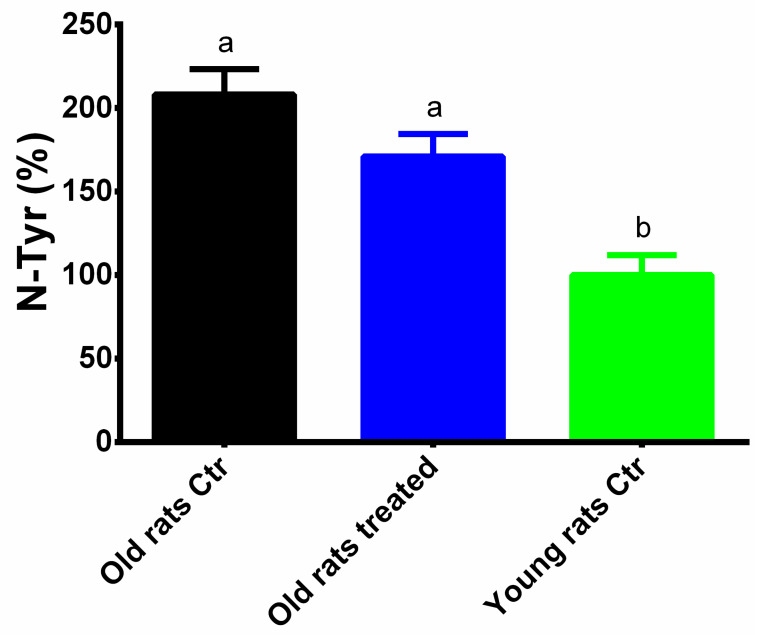
Levels of N-Tyr. Results are expressed as mean ± SEM, and *p* < 0.05 was considered statistically significant. Statistical significance was calculated by one-way ANOVA analysis. Different letters reveal significant differences. Old rats Ctr refers to old animals treated with placebo; Old rats treated refers to old animals treated with Taurisolo^®^; Young rats Ctr refers to young animals treated with placebo. **Abbreviations:** N-Tyr, nitrotyrosine.

Similarly, the chronic treatment with Taurisolo^®^ counteracts the increase in N-Tyr levels induced by ageing (208 ± 15.2% vs. 171 ± 14.3%, Old rats control vs. Old rats treated, *p* = 0.06; Young rats control: 100 ± 11.9%) (Figure 6).

### 3.5. Taurisolo^®^ Treatment Modulates the Expression of Oxidative Stress- and Inflammation-Related Genes in Muscle

As shown in Table 2, it was registered that old rats treated with the placebo had a reduced expression of antioxidant genes, including CAT, GPx, Sirt1 and Mn-SOD, and an increased pro-inflammatory genes expression, including IL-6 and -10, in comparison to the young animals. In contrast, we observed that chronic treatment with Taurisolo^®^ was able to counteract the negative influence of ageing on both OxS- and inflammation-related genes.

## 4. Discussion

Besides the historically known and well-established relationship between OxS and several diseases, many evidences reported a strong implication in the ageing processes. Indeed, the Free radical theory, postulated by Harman, highlights the crucial role played by OxS in senescence [2,3], suggesting that the pro-/antioxidant unbalance is a key factor in the development of several age-related pathological conditions, including muscle atrophy [7,8,9,10,11,12]. Finding novel approaches to contrast the age-related functional body decline is a major concern and, in this sense, polyphenol-based nutraceutical supplementation could be part of a program to combat age-related functional decline, in addition to the well-known beneficial effects of the physical activity.

In the present study, it has been shown that the ability of a novel nutraceutical formulation based on grape pomace polyphenolic extract (named Taurisolo^®^) ameliorates OxS-related biomarkers in the skeletal muscle of aged rats. The complex polyphenol profile [27] suggests a marked antioxidant potential of Taurisolo^®^, as previously demonstrated in a clinical trial on overweight/obese subjects reporting that eight-week treatment with Taurisolo^®^ significantly reduced the levels of OxS-related biomarkers, including trimethylamine-N-oxide (TMAO) and oxidized-LDL (oxLDL) [34]. Besides the potential involvement in biochemical processes, the intrinsic antioxidant activity of polyphenols may be primarily responsible for the exerted beneficial effects.

The first interesting result was the increased permanence time on the rotarod in aged rats treated with Taurisolo^®^. Although the rotarod test is generally used to evaluate the motor coordination mainly in aged brain-associated cognitive decline, we used these data to assess the effects of polyphenol treatment on muscle skills, eventually impaired by ageing. We reported that, after the 30-day treatment, old treated rats improved their ability to remain on the rotarod, registering no significant differences in comparison to the young group. On one hand, these results may suggest the possible ability of Taurisolo^®^ polyphenols to ameliorate the altered motor performance in aged rats, maybe though the increase of monoamine levels in specific brain regions involved in motor processes, including hippocampus and striatum [35,36]. On the other hand, it is plausible to speculate a direct improvement of muscular skills, as a result of the ameliorated muscle functionality. Previous studies reported the ability of polyphenols to improve exercise capacity and endurance in rats. According to previous studies, these effects may be due to role of polyphenols in both reductions of OxS in muscle and improvement of the vascular and endothelial function [37,38,39], resulting in a global amelioration of the skeletal muscle health.

The evidence reported in the literature leads to our belief that the antioxidant potential of Taurisolo^®^ might be at the basis of the effects observed both in vivo and in vitro. It is historically known that polyphenols exert their antioxidant activity through two major levels named (i) direct ROS-scavenging and modulation of the endogenous antioxidant defences and (ii) inhibition of both the metal-dependent production of free radicals and ROS-producing enzymes [40]. With respect to the first point, the scavenging activity of polyphenols is mainly due to their peculiar chemistry. Indeed, polyphenols are able to react with free radicals directly, including hydroxyl, superoxide, nitric oxide, alkoxyl and peroxyl radicals, through their benzene ring-bound hydroxyl groups, by which they transfer an electron to the ROS molecule, stabilizing the reactive species [41,42] and generating phenoxyl radicals that, in turn, react with a second radical forming a stable quinine structure [42]. In addition, due to their chemical features, polyphenols can directly counteract the production of ROS-chelating metal ions (mainly iron and copper), so preventing their participation in free radical formation reactions [40].

The present results evidenced a significant increase in total antioxidant capacity in muscles of aged rats, assessed by the FRAP assay. This test measures the ferric reducing ability of a sample and it is recognized as a useful method for assessing the antioxidant power [29]. Since the FRAP assay provides a putative index of reducing and antioxidant potential of biological samples, the results observed offer intriguing indications about the protective effect of chronic Taurisolo^®^ administration against OxS at the muscular level. Interestingly, it could be speculated that through the bloodstream, polyphenols or their metabolites, may reach the muscle [43,44] and exert in situ their antioxidant activity, counteracting the production of free radical by chelating iron ions, or inhibiting their reduction. This, in turn, provides a first important mechanism for the reduction of the OxS in muscle.

In addition to this direct activity of polyphenols in reducing OxS in muscle, a significant increase of the activity of key antioxidant enzymes, including GRd, GPx and CAT was also observed. These enzymes are part of the so-called endogenous antioxidant defence system acting via neutralizing free radicals [26]. In particular, CAT decomposes H_2_O_2_ in H_2_O before H_2_O_2_ reacts with metal ions generating hydroxyl radicals, while GRd and GPx regulate the maintenance of the balance between reduced and oxidized forms of glutathione (GSH) that acts as a free radical scavenging agent [26]. It has been previously reported that polyphenols are able to increase the activities of such antioxidant enzymes, including CAT, SOD and GPx [26]. In general, it can be speculated that the observed increase in antioxidant enzyme activities might be ascribed to both a direct effect of polyphenols in enhancing their enzymatic activities or an up-regulation of their expression. This up-regulation is mainly modulated via activation of the Keap1/Nrf2/ARE signalling pathway [40,45]. Another interesting signalling pathway involves Sirtuin 1 (Sirt1), an NAD^+^-dependent deacetylase playing a pivotal role in several biological processes, including ageing. Sirt1 modulates various nuclear factors, including forkhead box 0 (FOX0) and proliferator-activated receptor gamma coactivator 1α (PGC-1α), regulating the expression of antioxidant- and anti-ageing-related genes [26]. Additionally, in muscles, PGC-1 α stimulates the mitochondrial biogenesis activating the nuclear respiratory factor (NRF1) [26,46,47], representing a further protective mechanism contributing to muscle improvement. Polyphenols such as RSV have been demonstrated to indirectly activate Sirt1 through activation of the AMP-activated protein kinase (AMPK) [48]. The Sirt1-mediated regulation of FOX0 and PGC-1α results in enhanced expression of antioxidant genes, including SOD, GPx and CAT [49,50]. Interestingly, it has been reported that, in the gastrocnemius muscle of aged rats, the supplementation with red grape polyphenolic extract increased both the expression of PGC-1α and the activation of AMPK [1], providing further evidence for the involvement of polyphenols in these specific signalling pathways. In this sense, the observed enhanced Sirt1 expression in muscle of aged rats treated with Taurisolo^®^ might be responsible for an up-regulation of the antioxidant enzymes that, in turn, may explain the observed increased enzymatic activities of CAT, GPx and GRd. Moreover, it is possible that an enhanced mitochondriogenesis via PGC-1α activation explains the contrasted muscle decline, as shown by the increased permanence time of old rats treated on the rotarod, as an indicator of motor control. In contrast, evidence reported that polyphenols are able to suppress the nuclear factor kappa-light-chain-enhancer of the activated B cells (NF-κB) pathway that has been recognized as responsible for the increased expression of pro-oxidant and pro-inflammatory genes. More specifically, NF-κB is physiologically inactive in the cytosol by interaction with IκB. ROS and reactive nitrogen species (RNS) are stimuli causing the activation of NF-κB that translocates into the nucleus, promoting the expression of pro-inflammatory mediators/cytokines- and OxS-related genes [26]. In line with this evidence, our results demonstrated the ability of chronic Taurisolo^®^ administration to down-regulate the expression of pro-inflammatory genes, including IL6.

Moreover, protective effects of Taurisolo^®^ against both lipid and protein oxidation were observed, as it was shown by the reduced levels of MDA and N-Tyr, respectively. Lipid peroxidation is a direct consequence of OxS and it leads to both alteration of membrane biological function and cell damage [16]. In particular, ROS and RNS play a central role in promoting lipid peroxidation [26]. This is a chain-reaction process producing a large variety of reactive aldehydes, including MDA [51,52,53], which in turn, are stable and diffuse across the membranes attacking biomolecules in various sites, and causing biological and biochemical alterations leading to disease development [54,55,56]. In this sense, MDA can be considered as a marker of injury [57] and its levels have been found increased in sarcopenic subjects [16]. There is evidence about the activity of free radical scavenging agents to inhibit the lipid peroxidation, particularly polyphenols including RSV [58], due to its ability to donate hydrogen, generating phenoxyl radicals [59,60,61]. ROS and RNS are also involved in protein oxidation processes [26] leading to the formation of both protein carbonyls and advanced oxidation protein products [26] which have been found to be age-dependently increased in humans [62]. Particularly, among the various free radicals, RNS are implicated in reactions with amino acid side chains such as tyrosine, forming nitrogenated proteins (i.e., N-Tyr) [63]. Polyphenols have been demonstrated to efficiently counteract the ROS/RNS-mediated protein oxidation [26], contributing to the maintenance of cellular proteostasis [64]. Overall, the current results are in line with this evidence, confirming the antioxidant activity of Taurisolo^®^ at the muscular level and serve to speculate its beneficial potential in the protection against the direct repercussion of chronically increased OxS.

Our promising, observed results are in line with other studies investigating the beneficial effects of grape-derived polyphenols at a muscular level. Among these, a study conducted on a murine model of chronic high-grade inflammation (transforming growth factor [TGF] mice) demonstrated that four-week supplementation with red grape polyphenols significantly mitigated muscle atrophy, mainly improving the mitochondrial function [65]. Similarly, in IL10-knockout mice, the 12-week treatment with grape seed extract prevented muscle wasting [66]. Moreover, in diabetic rats, grape polyphenol supplementation reduced the OxS by up regulating the activities of antioxidant enzymes [67]. Interestingly, in vitro studies reported the ability of grape polyphenols in reducing the OxS in muscle cell lines, via reducing the levels of ROS and increasing the antioxidant defences [68,69,70,71], in particular modulating both the expression and the activities of antioxidant enzymes [72].

However, the present results have some limitations. Firstly, although the antioxidant potential of Taurisolo^®^ in muscle can be demonstrated by evaluating a number of OxS-related biomarkers, it cannot be directly measured the levels of ROS; nevertheless, the analysis performed indirectly allows us to consider that the global production of ROS in the muscle of aged rats treated with Taurisolo^®^ was reduced. In addition, no data regarding the muscle mass of study animals are available, so it cannot be concluded that the chronic treatment with Taurisolo^®^ exerts benefits in counteracting muscle atrophy. Nevertheless, the large body of evidence indicating the OxS as one of the major causes of muscle loss leads us to speculate about this potentially beneficial effect of Taurisolo^®^ polyphenols, although further studies are needed. Additionally, although previous studies reported that the chronic supplementation with polyphenols increased the activity of the manganese superoxide dismutase (MnSOD) both in aged mice [23] and rats [73] (via activating Sirt1 and subsequent FOXO-mediated MnSOD up-regulation), the present study only focused on determination of the combined Cu/Zn and MnSOD activities. Finally, concerning the observed body weight reduction in animals treated with Taurisolo^®^, although the well-established effect of polyphenol supplementation on weight and/or fat mass loss, we cannot provide any conclusion about the potential role of our nutraceutical in this sense, since no quantitative analyses have been performed on adipose tissue. On the other hand, as the main strength, this is the first study demonstrating the effect of a complex polyphenol formulation in reducing the OxS at muscular level and improving the muscle performance in aged animals. Interestingly, the dose administered in the animal experiments (100 mg/kg/day) is compatible with a possible human dose. Indeed, according to Reagan-Shaw and colleagues [74], the dose used is fewer than 1000 mg in human, which is considered by the Commission Regulation (EC) No. 258/1997 as the maximum safe polyphenolic extract daily amount compatible with a good health state.

## 5. Conclusions

**Figure 7:**
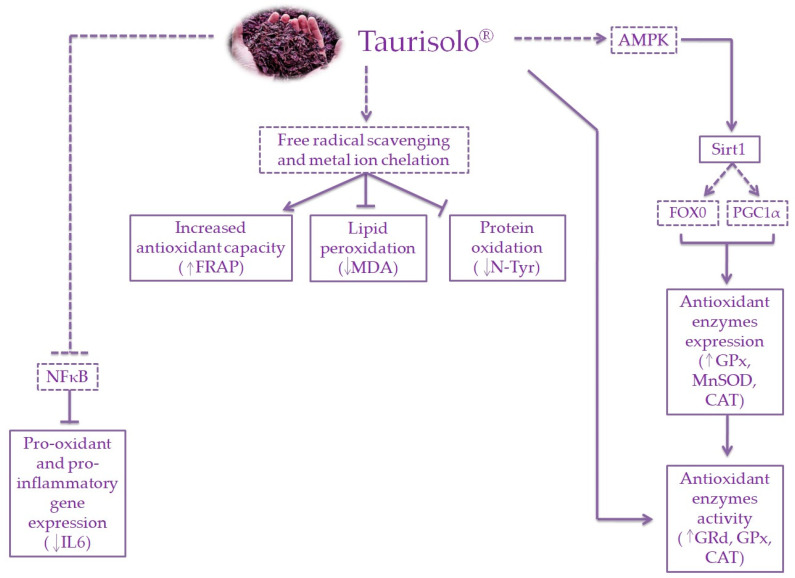
Schematic representation of the potential mechanisms of action of Taurisolo^®^ —indicates the mechanisms directly observed; **-----** indicates the mechanisms/pathways not directly observed, but reported in the literature.

In summary, the present study demonstrates that chronic treatment with Taurisolo^®^ significantly reduced the OxS and the oxidative damage at muscular level and improved the muscle performance in aged rats via improving the oxidative status at a muscular level. In particular, Taurisolo^®^ polyphenols have been demonstrated to (i) increase the total antioxidant capacity of muscles, (ii) increase the activities of antioxidant enzymes, (iii) reduce both the lipid and protein oxidation and (iv) modulate the expression of OxS- and inflammation-related genes (Figure 7). These results provide basic evidence driving the research toward further studies aimed to prove, in humans, the efficacy of this nutraceutical formulation in the management of age-related muscle impairments.

## Figures and Tables

**Table 1:** Primers sequences and Real-Time PCR conditions.

Gene	Sequence	Temperature (°C)
**Β-Actin**	Fw: **5**′-AGG GAAATCGTGCGTGAC-**3**′	95 °C 15 Seg 60 °C 30 Seg 72 °C 30 Seg
	Rev: **5**′-CGCTCATTGCCGATAGTC-3′	
**Mn-SOD**	Fw: **5**′-GGCCAAGGGAGATGTTACAA-**3**′	95 °C 15 Seg 60 °C 30 Seg 72 °C 30 Seg
	Rev: **5**′-GCTTGATAGCCTCCAGCAAC-**3**′	
**IL-10**	Fw: **5**′-GGCTCAGCACTGCTATGTTGCC-**3**′	95 °C 15 Seg 60 °C 30 Seg 72 °C 30 Seg
	Rev: **5**′-AGCATGTGGGTCTGGCTGACT-**3**′	
**IL-6**	Fw: **5**′-GCCACTGCCTTCCCTACTTCA-**3**′	95 °C 15 Seg 60 °C 30 Seg 72 °C 30 Seg
	Rev: **5**′-GACAGTGCATCGCTGTTCA-**3**′	
**GPx**	Fw: **5**′-GCTCATGACCGACCCCAAGT-**3**′	95 °C 15 Seg 65 °C 30 Seg 72 °C 30 Seg
	Rev: **5**′-GCCAGCCATCACCAAGCCAATA-**3**′	
**Catalase**	Fw: **5**′-TGGCCTCCGAGATCTTTTCAATG-**3**′	95 °C 15 Seg 63 °C 30 Seg 72 °C 30 Seg
	Rev: **5**′-GCGCTGAAGCTGTTGGGGTAGTA-**3**′	
**Sirt-1**	Fw: **5**′-TGGAGCAGGTTGCAGGAATCCA-**3**′	95 °C 15 Seg 60 °C 30 Seg 72 °C 30 Seg
	Rev: **5**′-TGGCTTCATGATGGCAAGTGGC-**3**′	

Abbreviations: Mn-SOD, mitochondrial superoxide dismutase; IL-10, interleukin-10; IL-6, interleukin-6; GPx, glutathione peroxidase; Sirt-1, sirtuin 1.

**Table 2:** Effect of Taurisolo^®^ chronic treatment on oxidative stress- and inflammation-related genes.

	Young Rats Ctr	Old Rats Ctr	Old Rats Treated
**CAT**	1.00 ± 0.36 ^a^	0.18 ± 0.04 ^b^	0.68 ± 0.32 ^a^
**GPx**	1.00 ± 0.19 ^ab^	0.68 ± 0.09 ^a^	1.47 ± 0.38 ^b^
**IL6**	1.00 ± 0.24 ^a^	4.84 ± 1.46 ^b^	1.20 ± 0.25 ^a^
**Sirt1**	1.00 ± 0.21 ^ab^	0.73 ± 0.13 ^a^	1.72 ± 0.17 ^ab^
**Mn-SOD**	1.00 ± 0.21	0.79 ± 0.06	1.58 ± 0.58
**IL10**	1.00 ± 0.16	1.44 ± 0.09	1.39 ± 0.19

Results are expressed as mean ± SEM, and *p* < 0.05 was considered statistically significant. Statistical significance was calculated by one-way ANOVA analysis. Different letters reveal significant differences.
